# TGFbeta Induces Binucleation/Polyploidization in Hepatocytes through a Src-Dependent Cytokinesis Failure

**DOI:** 10.1371/journal.pone.0167158

**Published:** 2016-11-28

**Authors:** Marco De Santis Puzzonia, Angela Maria Cozzolino, Germana Grassi, Francesca Bisceglia, Raffaele Strippoli, Giulia Guarguaglini, Franca Citarella, Benedetto Sacchetti, Marco Tripodi, Alessandra Marchetti, Laura Amicone

**Affiliations:** 1 Istituto Pasteur Italia-Fondazione Cenci Bolognetti, Department of Cellular Biotechnologies and Haematology, Sapienza University of Rome, Rome, Italy; 2 L. Spallanzani National Institute for Infectious Diseases, IRCCS, Rome, Italy; 3 Institute of Molecular Biology and Pathology, CNR National Research Council, c/o Department of Biology and Biotechnology, Sapienza University of Rome, Rome, Italy; 4 Department of Molecular Medicine, Sapienza University of Rome, Rome, Italy; University of Navarra School of Medicine and Center for Applied Medical Research (CIMA), SPAIN

## Abstract

In all mammals, the adult liver shows binucleated as well as mononucleated polyploid hepatocytes. The hepatic polyploidization starts after birth with an extensive hepatocyte binucleation and generates hepatocytes of several ploidy classes. While the functional significance of hepatocyte polyploidy is becoming clearer, how it is triggered and maintained needs to be clarified. Aim of this study was to identify a major inducer of hepatocyte binucleation/polyploidization and the cellular and molecular mechanisms involved. We found that, among several cytokines analyzed, known to be involved in early liver development and/or mass control, TGFbeta1 was capable to induce, together with the expected morphological changes, binucleation in hepatocytes in culture. Most importantly, the pharmacological inhibition of TGFbeta signaling in healthy mice during weaning, when the physiological binucleation occurs, induced a significant decrease of hepatocyte binucleation rate, without affecting cell proliferation and hepatic index. The TGFbeta-induced hepatocyte binucleation resulted from a cytokinesis failure, as assessed by video microscopy, and is associated with a delocalization of the cytokinesis regulator RhoA-GTPase from the mid-body of dividing cells. The use of specific chemical inhibitors demonstrated that the observed events are Src-dependent. Finally, the restoration of a fully epithelial phenotype by TGFbeta withdrawal gave rise to a cell progeny capable to maintain the polyploid state. In conclusion, we identified TGFbeta as a major inducer of hepatocyte binucleation both in vitro and in vivo, thus ascribing a novel role to this pleiotropic cytokine. The production of binucleated/tetraploid hepatocytes is due to a cytokinesis failure controlled by the molecular axis TGFbeta/Src/RhoA.

## Introduction

Polyploidy, a state in which a cell possesses more than two sets of homologous chromosomes, is a physiological feature of few mammalian cell types including skeletal and cardiac muscle cells, megakaryocytes and parenchymal liver cells [1]. Liver polyploidization is a process specifically occurring during the weaning period: at birth, mammals show a fully mononucleated liver tissue, whereas during the first post-natal months binucleated and mononucleated parenchymal cells with a higher content of DNA are produced [2, 3]. The binucleation of hepatocytes represents the first step of the polyploidization process and it has been demonstrated to be dependent on a cytokinesis failure [4]. In the adult, the majority of hepatocytes (about 85% in rodents and 40% in humans) is polyploid [5–7]. Interestingly, recent studies indicate polyploidy as a mechanism for stress-induced liver adaptation, during the response to xenobiotic/chemical/oxidative damage. Duncan and coworkers demonstrated that polyploid hepatocytes retain their capacity to respond to mitogenic stimuli giving rise to cell progeny with a higher DNA content (up to 16C) or aneuploid cells by multipolar mitosis [5]. Aneuploid hepatocytes can be subjected, upon different types of injury, to a darwinian selection, producing clones with specific karyotypes [8, 9]. Thus, it appears that polyploidy/aneuploidy is instrumental to liver function in allowing clonal expansion of hepatocytes genetically resistant to a specific injury.

Despite the interest derived from this stimulating evidence, how liver polyploidization is triggered and regulated during the weaning period remains an open question. Several data indicate the liver polyploidization as a complex multifactorial process dependent on intrinsic cell features and environmental cues, including soluble factors. In particular, in transgenic mouse model a delay in the establishment of hepatic polyploidy was associated with the overexpression of TGFalpha [10]. Furthermore, it has been shown that triiodothyronine positively regulates polyploidization in adult rat liver [11] and that high insulin blood levels increase the formation of binucleated/tetraploid hepatocytes via activation of the PI3K/AKT pathway [12, 13]. Many intrinsic regulators of hepatic polyploidy have been also described, including p53, E2F1, E2F8 and c-Myc [1].

In the current study, we demonstrated for the first time the role of TGFbeta1 in triggering hepatocyte binucleation/polyploidization. The biological relevance of these findings has been demonstrated in healthy growing mice, where TGFbeta activity resulted crucial for the formation of binucleated/tetraploid hepatocytes. With regard to cellular and molecular mechanisms, we showed that TGFbeta1 induces binucleation in hepatocytes through a cytokinesis failure associated with the delocalization from the midbody of RhoA-GTP (a well-known regulator of cytokinesis in mammalian cells) [14] and that these events are Src-dependent.

## Materials and Methods

### Cell lines and culture conditions

Hepatocyte cell lines used in this work (MMH/E14 and WT/3A) are standardly grown in RPMI (Gibco) medium supplemented with 10% FBS, 10μg/ml Insulin, 50ng/ml EGF and 30ng/ml IGF-2, on collagen I (Transduction Laboratories, Lexington, UK) coated dishes (Falcon-BD, Franklin Lakes, NJ, USA). MeT-5A lung mesothelial cell line (ATCC® CRL-9444™) is grown in DMEM (Gibco) supplemented with 10% FBS on plastic (Falcon-Bd Franklin Lakes, NJ, USA).

For treatments the media have been supplemented, where indicated, with 10ng/ml of TGFbeta1 (PeproTech Inc, Rocky Hill, NJ), 200ng/ml of Recombinant Murine Wnt3a (315–20, PeproTech Inc., Rocky Hill, NJ, USA), 100ng/ml FGF-1 of Recombinant Human Fibroblast Growth Factor Acidic (13241–013, Gibco, ThermoFisher Scientific Inc., MA, USA), 100ng/ml of FGF-2 Recombinant Mouse Fibroblast Growth Factor Basic (12343623, ImmunoTools GmbH, Germany), 2μM of Src inhibitor PP2 (Calbiochem, Merck Chemicals Ltd. Beeston, Nottingham, UK), 0.5μM of Src inhibitor SU6656 (Cayman Chemical, Ann Arbor, MI, USA), 10μM of PI3K inhibitor LY294002 (Calbiochem, Merck Chemicals Ltd. Beeston, Nottingham, UK) and 5μM of TGFβ receptor I/II inhibitor LY2109761 (Selleckchem,USA).

### Quantification of binucleation

Several optical microscope images of Giemsa stained cells were captured and analysed for binucleation. More than 1000 cells, in at least 20 random fields, were counted for each experimental condition and the values were expressed as percentage of binucleate cells over total cell number.

### Immunofluorescence

Cells were rinsed in PBS, fixed for 10 min with 4% formaldehyde in PBS and permeabilized 5 min with 0.1% Triton-X100. After fixation, coverslips were blocked in PBS containing 3% bovine serum albumin for 30 min at 37°C, before being processed for immunofluorescence.

Ki67 immunostaining on tissue slices has been performed following standard procedures.

Antibody dilution was as follows: anti-RhoA-GTP (NewEast Biosciences) 1:400; anti-Aurora B (AbCam ab3609) 1:100; anti-E-Cadherin (BD Biosciences Pharmingen, Bedford, MA) 1:50; anti-Snail (AbCam) 1:50; anti-Ki67 (BD Bioscience Pharmingen, USA; 1:25)

Secondary antibodies conjugated to Alexa-488 or Alexa-594 (Molecular Probes, Invitrogen, San Diego, CA; 1:400) were used as recommended by the supplier. Alexa Fluor 594 Phalloidin (a12381) (Thermo Fisher Scientific Inc., MA, USA) and Alexa Fluor 647–Phalloidin (from Invitrogen, Carlsbad, CA) were utilized for the staining of F-actin.

DNA was counterstained with 0.1 μg/ml 4'-6'-Diamidino-2-phenylindole (DAPI, Sigma-Aldrich, St Louis, MO).

All stained samples were analyzed under a Nikon Microphot-FXA microscope equipped with a CCD camera and digital images were acquired with Nikon NIS-elements software or using a Leica SP5 spectral confocal microscope.

### Western Blotting

Cells were lysed in RIPA buffer, separated by SDS–PAGE and transferred to nitrocellulose membranes following standard protocols. The membranes were incubated overnight with the following primary antibodies: anti-phospho-Src (Tyr416) (2101, 1:1000), anti-phospho-AKT (Ser473) (9271; 1:1000) and anti-phospho-SMAD3 (Ser423/425) (9520, 1:1000) (Cell Signalling Technology Boston, USA); anti–Tubulin (TU-02, 1:1000), and anti–CDK4 (c-22; 1:1000) (Santa Cruz Biotechnology, Inc., CA, USA). Blots were incubated with HRP-conjugated anti-rabbit and anti-mouse secondary antibodies (Bio-Rad Laboratories, Inc., Hercules, CA, USA). Immunoreactivity was detected by Enhanced Chemiluminescence reaction (WESTAR NOVA 2011, Cyanagen, Bologna, Italy) following the manufacturer’s instructions.

### Time-lapse video microscopy

TGFbeta1-treated or untreated hepatocytes were seeded on a glass-button collagen I coated micro-dish (Ibidi, Germany) and after 48 h placed on a micro-incubator stage (OKO Lab, Burlingame, CA) at 37°C and 5% CO2, for time-lapse analysis. Time-lapse video microscopy was performed using a Nikon Ti Eclipse microscope equipped with a 40X PlanFluor objective. Differential interference contrast images were acquired every 8 min for 24 h. The acquired image series was analyzed with NIS Elements (Nikon) and ImageJ (rsbweb.nih.gov/ij/) software.

### RNA extraction and RT–qPCR

Total RNA was extracted with NucleoSpin® RNA II kit (Macherey–Nagel, Germany) and reverse-transcribed with iScript cDNA Synthesis Kit (Bio-Rad Laboratories, Inc., Hercules, CA, USA). cDNA was amplified by qPCR reaction using BioRad Miniopticon with KAPA SYBR® Green FAST qPCR mix (KAPABIOSYSTEMS, Woburn, MA, USA). Primers: mouse E-cadherin forward 5′- CTACTGTTTCTACGGAGGAG -3′ and reverse 5′- CTCAAATCAAAGTCCTGGTC -3′; mouse Snail forward 5′- CCACTGCAACCGTGCTTTT -3′ and reverse 5′- CACATCCGAGTGGGTTTGG -3′.

### Ethics statement

Animal care was carried out in accredited facility and according to the criteria outlined in the ‘Guide for the Care and Use of Laboratory Animals’ of the National Academy of Sciences, published by the National Institutes of Health (NIH publication 86–23 revised 1985). All procedures were carried out in accordance with the Italian legislation governing the use of animals in experimentation (DL 116/92). No approval of ethics committee was required by Italian Law on the use of animals for scientific purposes in force during the period of the study. Animals were sacrificed under anesthesia by cervical dislocation to minimize suffering.

### Animal housing and treatment

Wild-type C57BL6 mice (Charles-River Laboratory) were maintained under temperature, humidity and specific-pathogen free conditions, and housed in standard polypropylene cages with *ad libitum* access to food and water. Four mice were treated by TbetaR inhibitor LY2109761 (Selleckchem) in 1% carboxymethyl cellulose (CMC) (Sigma), orally administrated by gavage (days 18–32 after birth), at the dose of 50 mg/kg twice a day (days 1–5 of each week). Four control mice received 1% CMC, administrated with the same procedure. Animal welfare was monitored daily. Mice were sacrificed at 32nd day and liver samples have been paraffin embedded and sectioned.

### Flow cytometry

Cells were suspended in Ca^++^/Mg^++^-free PBS and fixed with 50% glacial MetOH for 10 min. After washing with PBS the cells were incubated with 50μg/ml propidium iodide at 37°C for 30 min. DNA content was analyzed with the FACS Calibur (Becton Dickinson, Franklin Lakes, NJ, USA) and WinMDI 2.9 software.

### Statistical analysis

Generally, experiments were carried out with n ≥ 3. In all panels, data are presented as mean ±s.e.m. Statistical significance was determined using two-tailed Student t-test and p value<0.05 was considered statistically significant (p value<0.05 = *, p value<0.01 = ** and p value<0.001 = ***).

## Results

### TGFbeta1 induces binucleation in hepatocyte cell lines

To investigate the role of soluble factors in the induction of hepatocyte binucleation, we firstly performed a rough screening of the effects on hepatocyte binucleation of several molecules known to be involved in early liver development and/or mass control [15]. As cell model we utilized the well differentiated, non-tumorigenic hepatocyte cell line MMH/E14, derived from fetal livers of cyto-Met transgenic mice at 14,5 days post-coitum, largely characterized and utilized in several studies of liver functions [16–21]. MMH/E14 cells are mostly mononucleated, with a stable and homogeneous chromosome number (data not shown). In a single experiment, MMH/E14 cells were independently stimulated with EGF, IGF-2, Wnt3a, TGFbeta1, FGF-1 and FGF-2, and analyzed after 72 hours for binucleated cell content.

**Fig 1:**
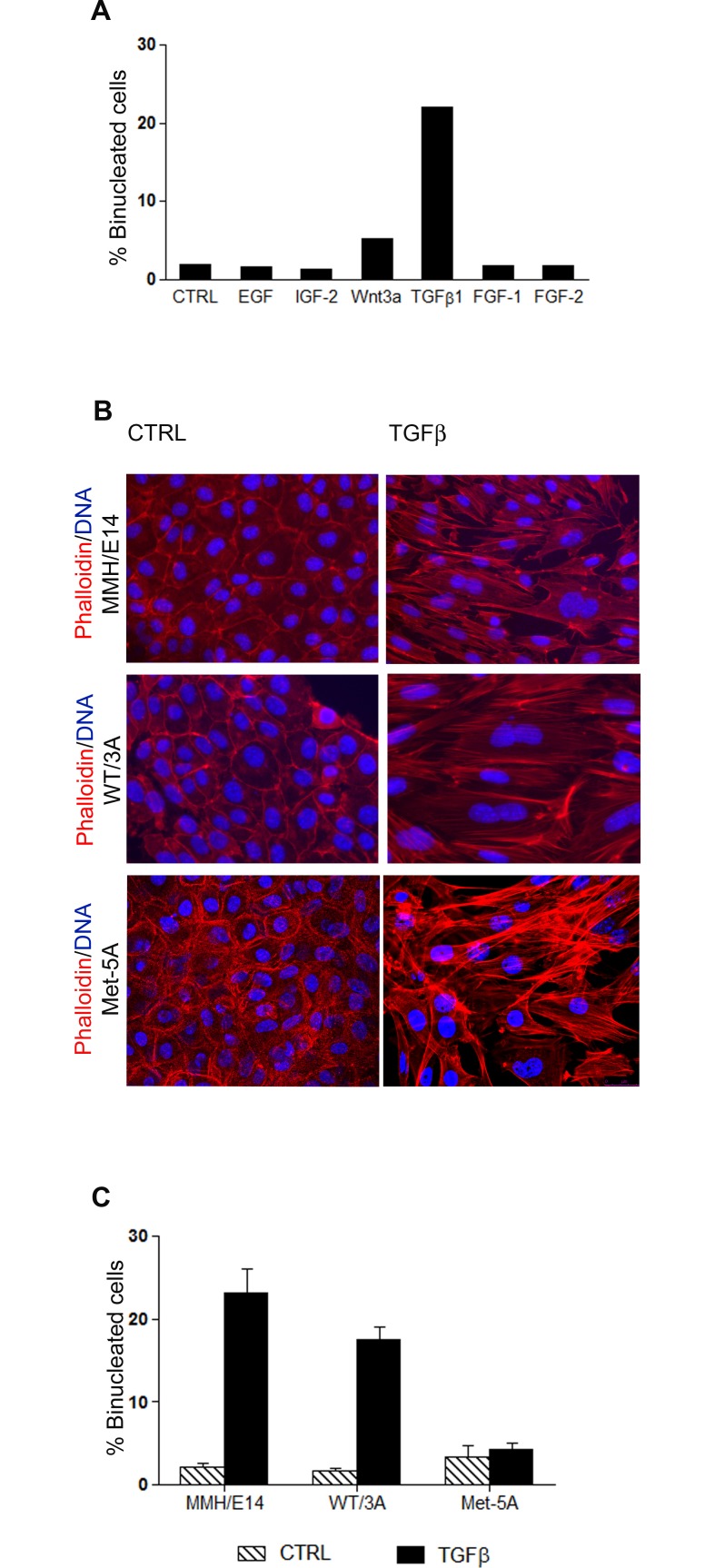
TGFbeta1 induces hepatocyte binucleation. (A) Percentage of binucleated cells in cultures of MMH/E14 cells maintained in RPMI w/o additional growth factors for three weeks and then supplemented with the indicated cytokines and growth factors for 72h. (CTRL = untreated cells). (B) Immunofluorescence of untreated and TGFbeta1-treated MMH/E14 and WT/3A hepatocytes, and pleural mesothelial MeT-5A cell line after staining with phalloidin (red) and DAPI (blue). Images of MeT-5A cells was acquired by confocal microscopy. (C) Percentage of binucleated cells in cultures of hepatocyte cell lines (MMH/E14 and WT/3A) and of pleural mesothelial (MeT-5A) cell line, after 72 h of TGFbeta1 treatment; data are expressed as average values of four independent experiments, ± s.e.m.

Interestingly, among the tested cytokines, TGFbeta1 induced, together with a morphological epithelial-to-mesenchymal transition (EMT) as expected and previously described [22–24], also a weighty rate of binucleation compared to untreated cells (23,3±3% in TGFbeta1-treated versus 2,1±1,8% in untreated cells) (Fig 1A, Fig 1B and S1 Fig, upper panels, and Fig 1C).

A confirm of these results has been obtained in a second hepatocyte cell line, WT/3A (Fig 1B and S1 Fig, middle panels, and Fig 1C), derived from fetal livers of wild-type mice at 14,5 days post-coitum, and previously characterized [25, 26].

On the contrary, the non-tumorigenic pleural mesothelial MeT-5A cell line, similarly responsive to TGFbeta1 with respect to EMT [27], did not respond to the cytokine with formation of binucleated cells (Fig 1B and S1 Fig, lower panels, and Fig 1C), thus suggesting the binucleation as a possible hepatocyte-specific effect of TGFbeta.

### The inhibition of TGFbeta receptor hampers hepatocyte binucleation *in vivo*

**Fig 2:**
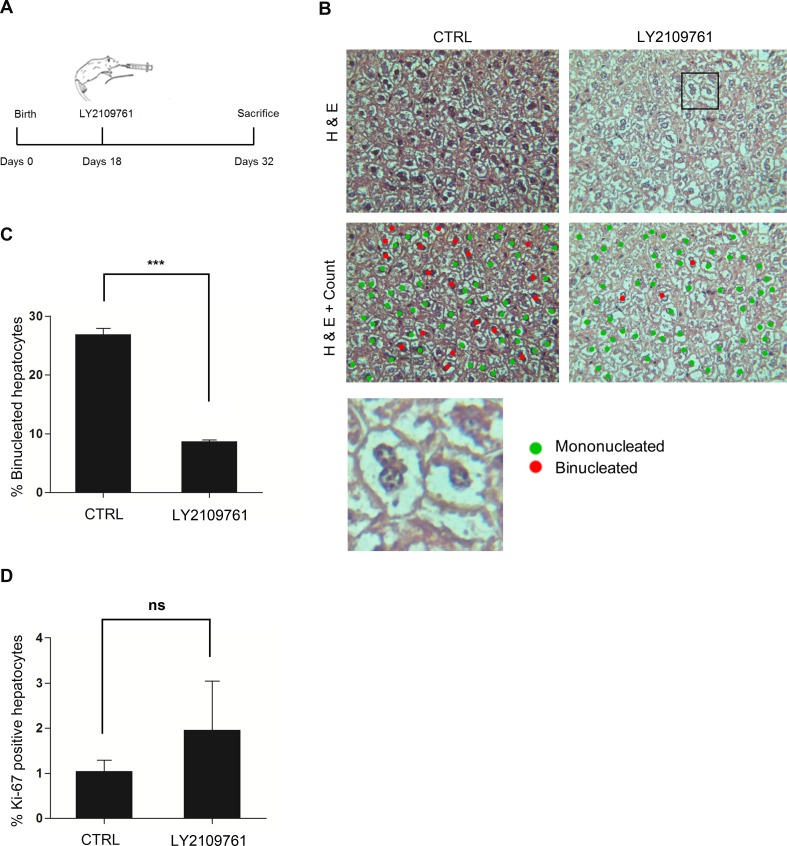
The *in vivo* inhibition of TGFbeta receptor hampers hepatocyte binucleation. (A) Experimental schedule for *in vivo* TGFbetaRI/II inhibitor (LY2109761) administration. (B) H&E of liver tissue from LY2109761-treated and control animals. In the lower panels are shown micrographs where binucleated and mononucleated cells are marked in red and green, respectively. A magnification of cells from the upper right panel was shown. (C) Percentage of binucleated cells of LY2109761-treated and control livers. Data are expressed as average values of counts of at least ten slices for each of four mice ± s.e.m. (number of counted cells: >1000 for each mouse). (***p value < 0.001). (D) Percentage of Ki-67 positive hepatocytes, analyzed by immunofluorescence on liver slices from LY2109761-treated and control mice. Data are expressed as average values of counts of at least five slices for each of four mice ± s.e.m. (number of counted cells: >400 for each mouse). (n.s. = not significant p value).

To challenge the *in vivo* significance of the results obtained in cultured hepatocytes, we analyzed straightaway the role of TGFbeta1 in liver binucleation/polyploidization of C57BL6 wild-type mice. To this end, we treated animals with LY2109761, a specific inhibitor of both TGFβ receptor type I and II (TGFbetaRI/II), whose pharmacodynamics/pharmacokinetics and toxicity have been well characterized [28]. After a preliminary experiment on WT/3A hepatocytes, in which we verified the efficacy of the inhibitor to impair the TGFbeta signaling (analyzing the level of phosphorylated Smad3) and the binucleation rate (S2A and S2B Fig respectively, lanes 1–4), LY2109761 was administered to mice as described in Fig 2A. The treatment timing and the dosage were in accord to previous works reporting the weaning period as the frame time in which binucleation occurs [2, 3] and 100mg/Kg as the daily dose able to inhibit the TGFbeta signaling in vivo [28–30].

At 32^nd^ day, mice were sacrificed and liver tissue analyzed for mononucleated and binucleated hepatocytes. LY2109761-treated mice showed a strong reduction of binucleated cells compared to the control mice (8,7 ±0,23% versus 26,9 ± 0,99%; P<0,001) (Fig 2B and 2C), thus demonstrating that the formation of binucleated hepatocytes *in vivo* requires the activity of TGFbeta pathway. Finally, in order to rule out that the reduced hepatocyte binucleation rate in LY2109761-treated mice was due to a decrease in cellular proliferation, liver slices were assessed by Ki67 staining. As shown in Fig 2D, livers from LY2109761-treated and control mice showed no significant difference in percentage of Ki67 positive cells, revealing that the drug administration did not affect the hepatocyte proliferation.

In accord with this result, and despite the total weight of the animals was slightly affected by the treatment (control mice: 17,1 g. ± 0,76, LY-treated mice: 14,8 g. ± 1,52), the liver weight/body weight ratio (0,042) was maintained.

These *in vivo* data strongly support the conclusion that TGFbeta is an inducer of hepatocyte binucleation and that it plays a crucial role in regulating the physiological liver binucleation/polyploidization *in vivo*.

### The hepatocyte binucleation results from a Src-dependent failure of cytokinesis

**Fig 3:**
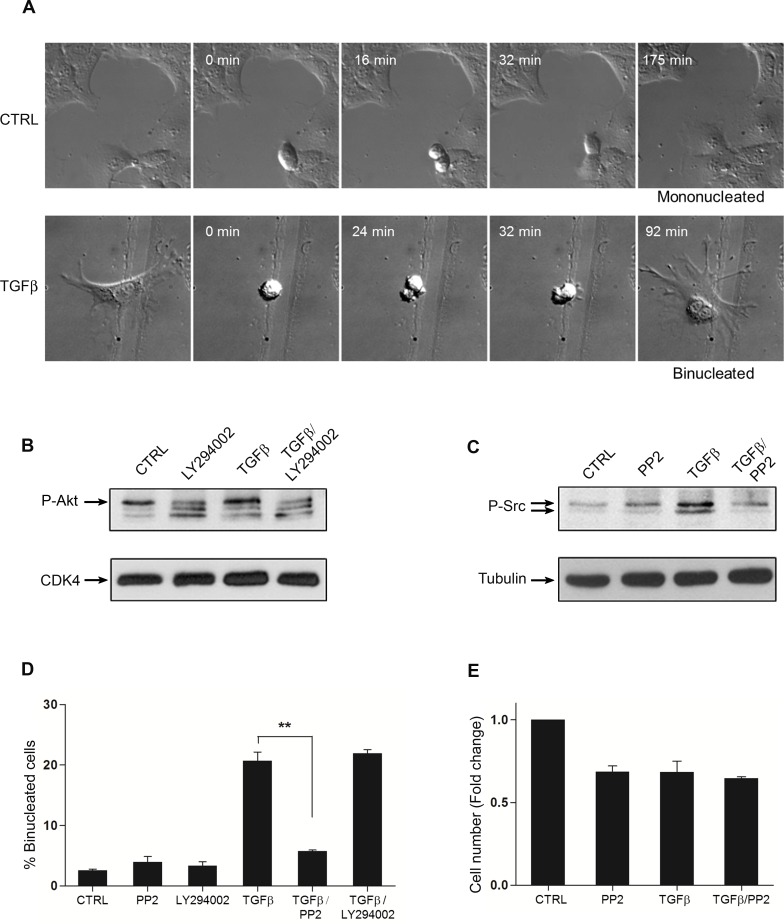
The hepatocyte binucleation results from a Src-dependent failure of cytokinesis. (A) Time-lapse video microscopy of single untreated (upper) and TGFbeta1-treated (lower) MMH/E14 hepatocytes. The dynamics of mitosis are compared and the cytokinesis failure in a single TGFbeta1-treated cell is shown in the last frame of the lower panel. (B) Western Blot analysis of the phosphorylated form of AKT (upper panel), target of PI3K, in the presence of TGFbeta1 with or without LY294002 (PI3K inhibitor, 10μM). CDK4 and tubulin were utilized as loading control. (C) Western Blot analysis of the phosphorylated form of Src kinase (upper panel) in the presence of TGFbeta1 with or without PP2 (Src inhibitor, 2μM). Src activity was analysed as autophosphorylation. Tubulin was utilized as loading control. (D) Percentage of binucleated cells in TGFbeta1-treated or untreated cultures in the presence or absence of the PI3K inhibitor, LY294002, or the Src inhibitor, PP2. Data are expressed as average values of three different experiments ± s.e.m. (**p value < 0.01). (E) Proliferation assay performed by cell count after 72 h of culture in the shown conditions. Data are expressed as average values of three different experiments ± s.e.m., and plotted as ratio between treated and untreated (CTRL = 1) cells.

In physiological and pathological conditions, different mechanisms are known to contribute to the formation of binucleated/polyploid cells [31]. With respect to the liver, the primary mechanism leading to binucleation is the cytokinesis failure [4]. In order to analyze the dynamics of mitosis during TGFbeta-mediated hepatocyte binucleation, a time lapse video microscopy of single mononucleated cells, with or without TGFbeta1, has been performed. Video analysis revealed that the treated cells progressed throughout the different phases of mitosis, up to an incomplete cytokinesis, when a regression of the cleavage furrow has allowed the formation of a single binucleated daughter cell (Fig 3A, S1 and S2 Movies). Of note, we were able to capture events of cytokinesis failure only in cultures of TGFbeta1-treated cells.

TGFbeta1 is well known to activate multiple downstream pathways. Among these, both PI3K and Src kinase signaling pathways have been found to mediate cytoskeleton rearrangement, which is fundamental for completion of cytokinesis [32]. In particular, PI3K is involved in the induction of the cytokinesis program in hepatocytes [13], whereas Src is required for cleavage furrow progression [33]. To elucidate the molecular mechanism of TGFbeta-mediated cytokinesis failure, resulting in the observed hepatocyte binucleation, pharmacological inhibitors of PI3K (LY294002) and Src (PP2) have been used on MMH/E14 cell line; the inhibitors’ efficacy has been verified by analysing the phosphorylated form of AKT and the auto-phosphorylated form of Src, respectively (Fig 3B and 3C). Whereas the inhibition of PI3K did not affect the number of binucleated cells, inhibition of Src largely hindered the binucleated phenotype observed in TGFbeta1-treated cells (Fig 3D). Of note, in our experimental condition (namely the low dose of PP2), the inhibition of Src did not affect the rate of proliferation of TGFbeta1-treated cells (Fig 3E).

The role of Src in TGFbeta-induced hepatocyte binucleation has been confirmed in WT/3A cells, also by means of a second Src inhibitor, SU6656 [34]. The efficacy of this inhibitor and its effects on binucleation are illustrated in S2B (lanes 5–6) and S2C Fig.

Altogether these results indicate that the mechanism by which TGFbeta1 induces hepatocyte binucleation requires Src kinase activity.

Interestingly, while we previously described the involvement of Src in some aspects of EMT [35, 36], low dose of Src inhibitors did not impede morphological changes and transcriptional regulation of Snail (EMT master regulator) and E-Cadherin (epithelial marker) (S3 Fig). Accordingly, the SMAD signaling pathway from which depends the switch-off of the epithelial program during TGFbeta-induced EMT in hepatocytes [37], resulted unaffected by Src inhibitor treatments (S2A Fig, lanes 5–8). This result suggests a role of Src in hepatocyte binucleation independent from EMT.

### The TGFbeta-induced Src activity is required for RhoA GTPase delocalization from the midbody

**Fig 4:**
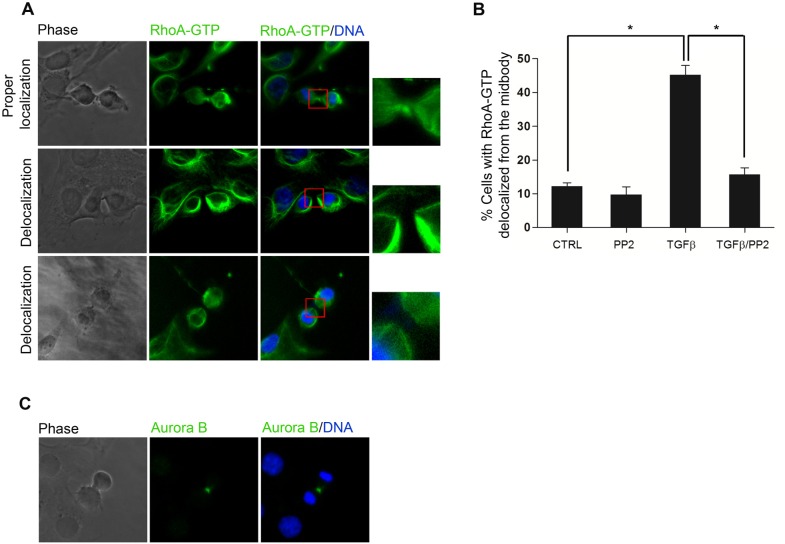
The TGFbeta1-induced cytokinesis failure is associated with the delocalization of active RhoA from the midbody. (A) Representative immunofluorescence of cytokinesis events with RhoA-GTP properly localized at the midbody (upper panel) or delocalized (middle and lower panels), considered for the count in both untreated hepatocytes and in TGFbeta-treated ones. In particular, the images refer to TGFbeta-treated hepatocytes. (B) Percentage of mitotic cells negative for RhoA-GTP signal at the midbody. Data are expressed as average values of three different experiments ± s.e.m (number of counted cells: >100 for each experimental condition) (*p value < 0.05). (C) Representative immunofluorescence of the cytokinesis events analysed in three independent experiments, showing the proper localization of Aurora B at the midbody upon TGFbeta1 treatment.

RhoA, a member of Rho GTPase family responsible for actin cytoskeleton rearrangements, is known to be necessary for cytokinesis completion, acting at the midbody during cleavage furrow ingression [14, 38]. In particular, the actin-myosin ring, required for a correct cytokinesis, is assembled at the equatorial cell cortex in a manner dependent on RhoA activity [39, 40]. As described for other cell types [41, 42], also in hepatocytes that fail the cytokinesis, the activation of RhoA pathway is impaired [4]. Furthermore, RhoA has been shown to be inactivated by Src kinase [43]. Starting from all these observations we investigated the involvement of RhoA in the Src-dependent binucleation induced by TGFbeta1. To this aim we analyzed by immunofluorescence the presence/localization of the GTP-bound active form of RhoA (Fig 4A) and found a higher percentage of cytokinesis figures with a delocalization of the protein from the midbody in TGFbeta-treated cultures compared to the control cultures (Fig 4B). Conversely, the percentage of cells with RhoA-GTP delocalization in double treated TGFbeta/PP2 cultures was comparable to the control, thus demonstrating that the displacement of active RhoA from the midbody associated to (and most likely responsible for) the binucleation induced by TGFbeta, is Src-dependent. Interestingly, in TGFbeta-treated cultures we could not find any cell showing delocalization from the midbody of Aurora B kinase, another cytokinesis regulator able to target a number of proteins to the cleavage furrow [44, 45] (Fig 4C shows a representative micrograph of proper localization of Aurora B observed both in untreated and in TGFbeta-treated cells). This result indicates a specific effect of the Src activity induced by TGFbeta1 on RhoA.

Overall, these data strongly support the conclusion that the TGFbeta/Src/RhoA axis is responsible for binucleation in hepatocytes.

### Binucleated hepatocytes recover epithelial morphology after TGFbeta withdrawal and proliferate maintaining the poliploidy state in their progeny

**Fig 5:**
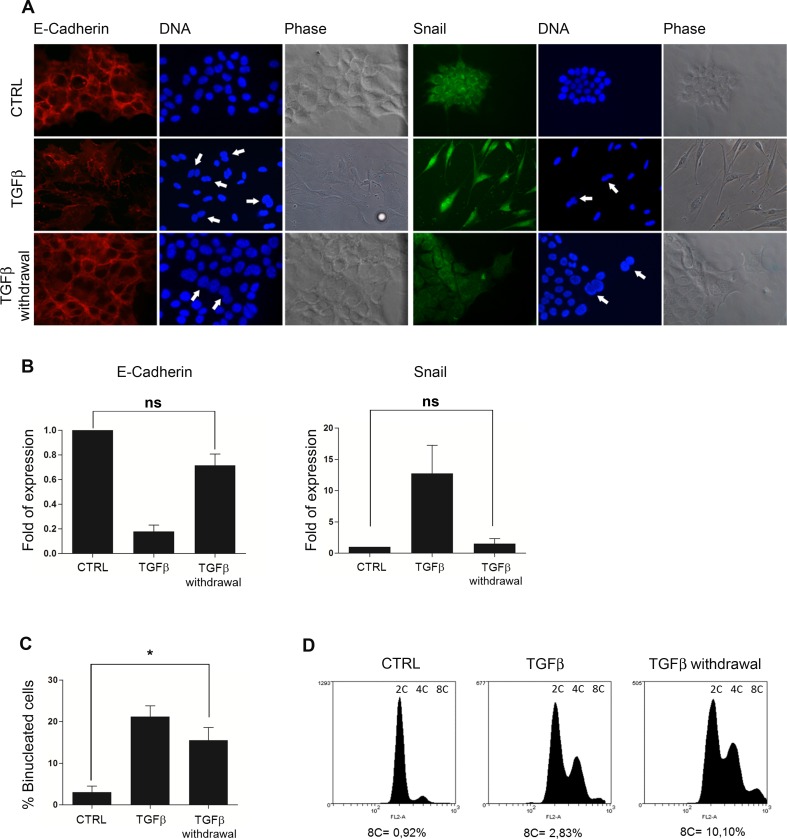
Tetraploid hepatocytes proliferate maintaining the poliploidy state in their progeny after TGFbeta1 withdrawal. (A) Immunofluorescence for E-Cadherin and Snail of TGFbeta1-treated, TGFbeta1-released and control MMH/E14 hepatocytes. Arrows indicate binucleated cells. (B) Transcriptional analysis by qRT-PCR of E-Cadherin and Snail in the indicated experimental conditions. Data are expressed as average values of three different experiments ± s.e.m. (ns = not significant p value), and plotted as ratio between treated and untreated (CTRL = 1) cells. (C) Percentage of binucleated cells in the indicated experimental conditions. Data are expressed as average values of three different experiments ± s.e.m. (*p value < 0,05). (D) Flow cytometry analysis for DNA content of cells cultured in the indicated experimental conditions.

As mentioned above, TGFbeta1 treatment induced hepatocyte binucleation together EMT. We previously reported that the TGFbeta-induced EMT is reversible into a Mesenchymal-to-Epithelial Transition (MET) upon TGFbeta withdrawal [24, 35]. Starting from the assumption that polyploid hepatocytes in the liver show a fully epithelial phenotype/differentiation, we investigated if cells reacquiring an epithelial identity by MET, are able to maintain in their cell progeny the polyploid state. To this end, cells treated with TGFbeta1 for 72 h were released for further 72 h. The analysis of cell morphology and of epithelial/mesenchymal markers confirmed that MET occurred in TGFbeta-released cells: (i) the cells reacquired an epithelial-like shape (Fig 5A); ii) E-Cadherin appeared localized at the cell membrane and Snail delocalized from the nucleus (Fig 5A) and (iii) mRNA levels of E-Cadherin and Snail were comparable to those of untreated control cells (Fig 5B).

The recovered epithelial population was then assessed for binucleated cell content: as shown in Fig 5C, epithelial TGFbeta-released cultures retained the fraction of binucleated cells (also indicated by arrows in Fig 5A). Interestingly, flow cytometry analysis of hepatocytes that have undergone MET, revealed the increase of the population with 8C DNA content, which was virtually absent in the untreated cells and barely represented in TGFbeta-treated cells (Fig 5D). Because of the inability to discriminate between tetraploid cells in G2 and octaploid cells in G1 with this analysis, the possibility that the 8C cells could be derived also from additional cytokinesis failure events needs further investigation.

Overall, these data demonstrate that binucleated/polyploid epithelial hepatocytes are able to proliferate and possibly to generate a fraction of cells with higher ploidy levels.

## Discussion

In this study we reported the new role of TGFbeta signalling in controlling hepatocyte cell division. We have demonstrated that TGFbeta1 triggers the formation of binucleated cell in hepatocyte cell lines and that the activity of TGFbeta pathway is mandatory for physiological hepatocyte binucleation *in vivo*. Observations made inhibiting the TGFbeta pathway in animals are of particular relevance since they permitted to highlight the role played by the cytokine in the healthy growing liver. So far, indeed, the role of TGFbeta in the liver had been widely explored in pathological conditions (cancer, fibrosis, compensatory regeneration and control of liver mass under mitogenic stimulus) [46–49], while data on its contribution to physiological processes are quite poor. Regarding the physiological postnatal liver polyplodization, TGFbeta can act in parallel with (or integrating) other signals, already described in the hepatic ploidy control. Recent observations, indeed, unveiled the crucial role played by TGFbeta1 in the stimulation of pancreatic beta cells, eventually resulting in the control of insulin level [50]. Since changes in insulin signaling, occurring at weaning in an AKT-dependent manner, were shown to be causal for hepatocyte cytokinesis failure [13], we can speculate that TGFbeta could act *in vivo* both directly on hepatocytes and indirectly through the induction of insulin by pancreatic beta cells. Further studies will allow to verify this hypothesis. In addition, conditions causing profound perturbation of liver homeostasis known to increase liver ploidy, such as partial hepatectomy [51, 52], iron overload [53] and oxidative injury [54, 55], are associated to an increase of TGFbeta expression/activity. In particular, TGFbeta is known to orchestrate multiple events during liver regeneration after partial hepatectomy [56]. Moreover, an increased expression of TGFbeta was observed in hepatic acinar zone 1 of rats with iron overload [57]; in this pathological condition the cytokine induces the expression of hepcidin, a protein involved in iron level control [58]. Similarly, TGFbeta increases in conditions of oxidative stress, where it mediates an Unfolded Protein Response [59]. All these literature data allow to speculate that TGFbeta could be directly responsible also for the polyploidy observed in these pathological conditions. Further investigations are required to validate this hypothesis.

With respect to the cellular mechanisms, we showed that, upon TGFbeta1 stimulation, cultured hepatocytes (that spontaneously give rise to diploid mononucleated progeny) fail cytokinesis through cleavage furrow regression, producing binucleated daughter cells. Our results, demonstrating that cytokinesis failure responsible for the formation of binucleated cells in response to TGFbeta1 is Src-dependent, integrate other works showing a role of Src activity in cytoskeleton rearrangement and in cytokinesis [43, 60]. Constitutive Src activity, indeed, produces delocalization of cytokinesis regulators such as MLKP1, Aurora B and INCENP from the midbody, resulting in the formation of multinucleated polyploid cells [33]. MLKP1 is known to be necessary for localization of RhoA at the midbody, where both are fundamental for cytokinesis completion [14]. Consistently with these data, we observed a Src-dependent delocalization of active RhoA from the midbody in TGFbeta-treated cells.

Finally, here we show that TGFbeta1 induced in hepatocytes a morphological and reversible EMT, as previously reported [24, 35]. Interestingly, the epithelial cells, reverted from mesenchymal cells upon TGFbeta1 withdrawal, maintained the ability to proliferate and to produce 4C progeny and, differently from the TGFbeta1-treated cells, also 8C cells. These data indicate that, while TGFbeta1 triggers the binucleation/polyploidization, it is not necessary for its maintenance. Mechanisms allowing cytokinesis failure of epithelial cells, and driving them into further level of ploidy when “primed” by TGFbeta, require further investigation.

Furthermore, these results, together with the here showed observation that inhibition of Src activity interfered with binucleation without affecting EMT process, suggest that EMT/MET and cytokinesis failure are uncoupled processes in triggering liver polyploidy. Actually, the possibility that EMT occurs in the liver both in pathological (i.e. fibrosis) or physiological (development and postnatal growing) conditions still remains a controversial issue.

**Fig 6:**
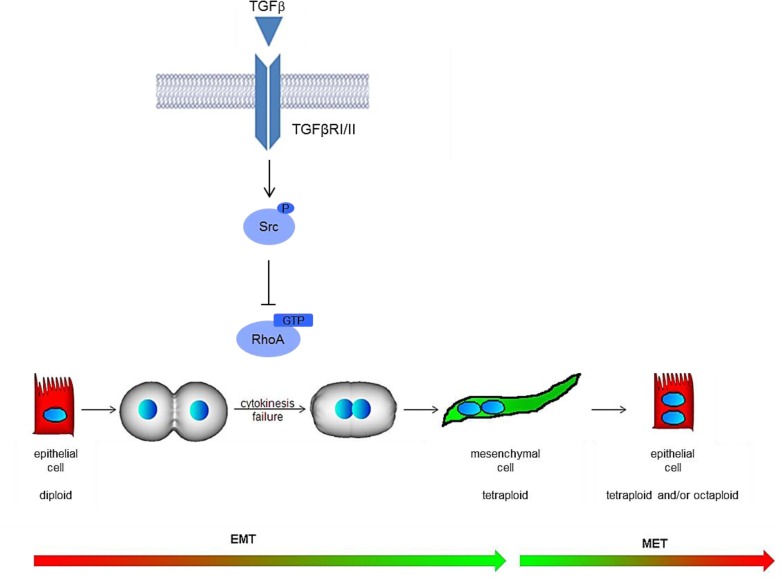
Graphical representation of the proposed TGFbeta-induced mechanism of hepatocyte binucleation.

In conclusion, we demonstrated a key role of TGFbeta in the regulation of hepatocyte binucleation and identified in the cytokinesis failure controlled by the molecular axis TGFbeta/Src/RhoA the mechanisms involved (Fig 6).

## Supporting Information

S1 Fig Optical microscope analysis of TGFbeta1-induced hepatocyte binucleation. Optical micrographs of Giemsa-stained untreated (epithelial) and TGFbeta1-treated (fibroblastoid) MMH/E14, WT/3A and Met-5A cell lines.
(PPTX)

S2 Fig Effects of the pharmacological treatments in WT/3A hepatocytes. (A) Western Blot analysis of the phosphorylated form of SMAD3 (upper panel), in the presence of TGFbeta1 with or without Src inhibitors (PP2, 2μM; SU6656, 0.5μM) or TGFbeta RI/II inhibitor (LY2109761, 5μM). CDK4 was utilized as loading control. (B) Percentage of binucleated cells in cultures of WT/3A hepatocytes treated as in (A). (C) Western Blot analysis of the phosphorylated form of Src kinase in the presence of TGFbeta1 with or without SU6656 (Src inhibitors, 0.5μM). Src activity was analysed as autophosphorylation, according to other reports (Kong et al., 2011). CDK4 were utilized as loading controls.
(PPTX)

S3 Fig Effect of PP2 treatment on EMT. (A) Immunofluorescence for E-Cadherin of MMH/E14 hepatocytes in the indicated experimental conditions. Arrows indicate the binucleated cells. (B) Transcriptional analysis by qRT-PCR of E-Cadherin and Snail of MMH/E14 hepatocytes in the indicated experimental conditions. Data are expressed as average values of three different experiments ± s.e.m., and plotted as ratio between treated and untreated (CTRL = 1) cells.
(PPTX)

S1 Movie Untreated hepatocytes undergo normal mitosis. Time lapse video microscopy showing a representative untreated mononucleated hepatocyte undergoing normal mitosis.
(MPG)

S2 Movie TGFbeta1-treated hepatocytes undergo cytokinesis failure. Time lapse video-microscopy showing a representative TGFbeta1-treated mononucleated hepatocyte undergoing cytokinesis failure and producing one binucleated cell.
(MPG)
