# Supplementary figures and images for: TGFbeta Induces Binucleation/Polyploidization in Hepatocytes through a Src-Dependent Cytokinesis Failure
Source: PLoS One. 2016 Nov 28;11(11):e0167158. doi: 10.1371/journal.pone.0167158 (PMC5125678; doi:10.1371/journal.pone.0167158)

## Slide 1
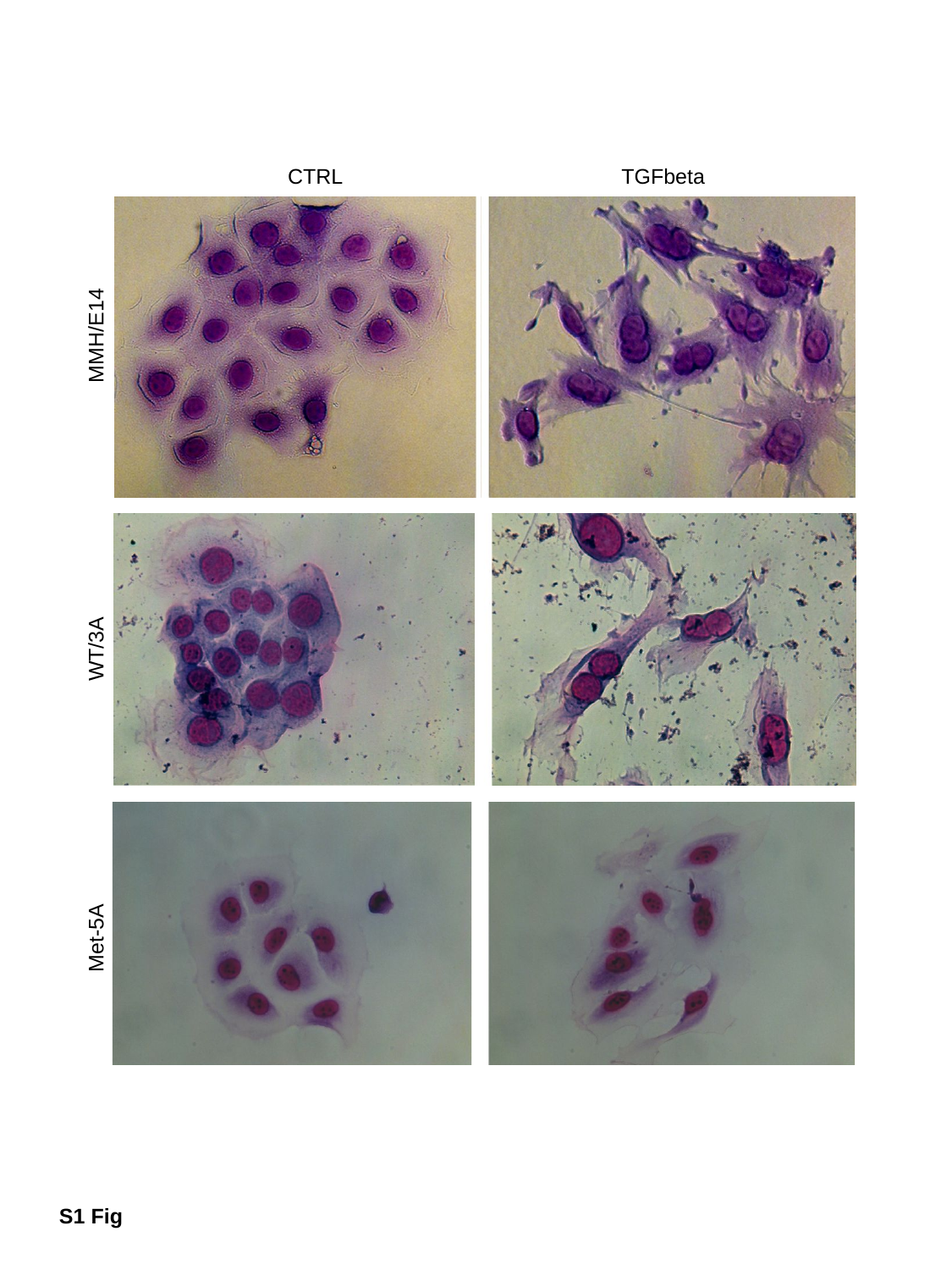

CTRL
TGFbeta
MMH/E14
WT/3A
Met-5A
S1 Fig

Supplement: S1 Fig — Optical micrographs of Giemsa-stained untreated (epithelial) and TGFbeta1-treated (fibroblastoid) MMH/E14, WT/3A and Met-5A cell lines. (PPTX) [file pone.0167158.s001.pptx]
