# Supplementary material for: TGFbeta Induces Binucleation/Polyploidization in Hepatocytes through a Src-Dependent Cytokinesis Failure
Source: PLoS One. 2016 Nov 28;11(11):e0167158. doi: 10.1371/journal.pone.0167158 (PMC5125678; doi:10.1371/journal.pone.0167158)
Supplement: S2 Fig — (A) Western Blot analysis of the phosphorylated form of SMAD3 (upper panel), in the presence of TGFbeta1 with or without Src inhibitors (PP2, 2μM; SU6656, 0.5μM) or TGFbeta RI/II inhibitor (LY2109761, 5μM). CDK4 was utilized as loading control. (B) Percentage of binucleated cells in cultures of WT/3A hepatocytes treated as in (A). (C) Western Blot analysis of the phosphorylated form of Src kinase in the presence of TGFbeta1 with or without SU6656 (Src inhibitors, 0.5μM). Src activity was analysed as autophosphorylation, according to other reports (Kong et al., 2011). CDK4 were utilized as loading controls. (PPTX) [file pone.0167158.s002.pptx]

## Slide 1
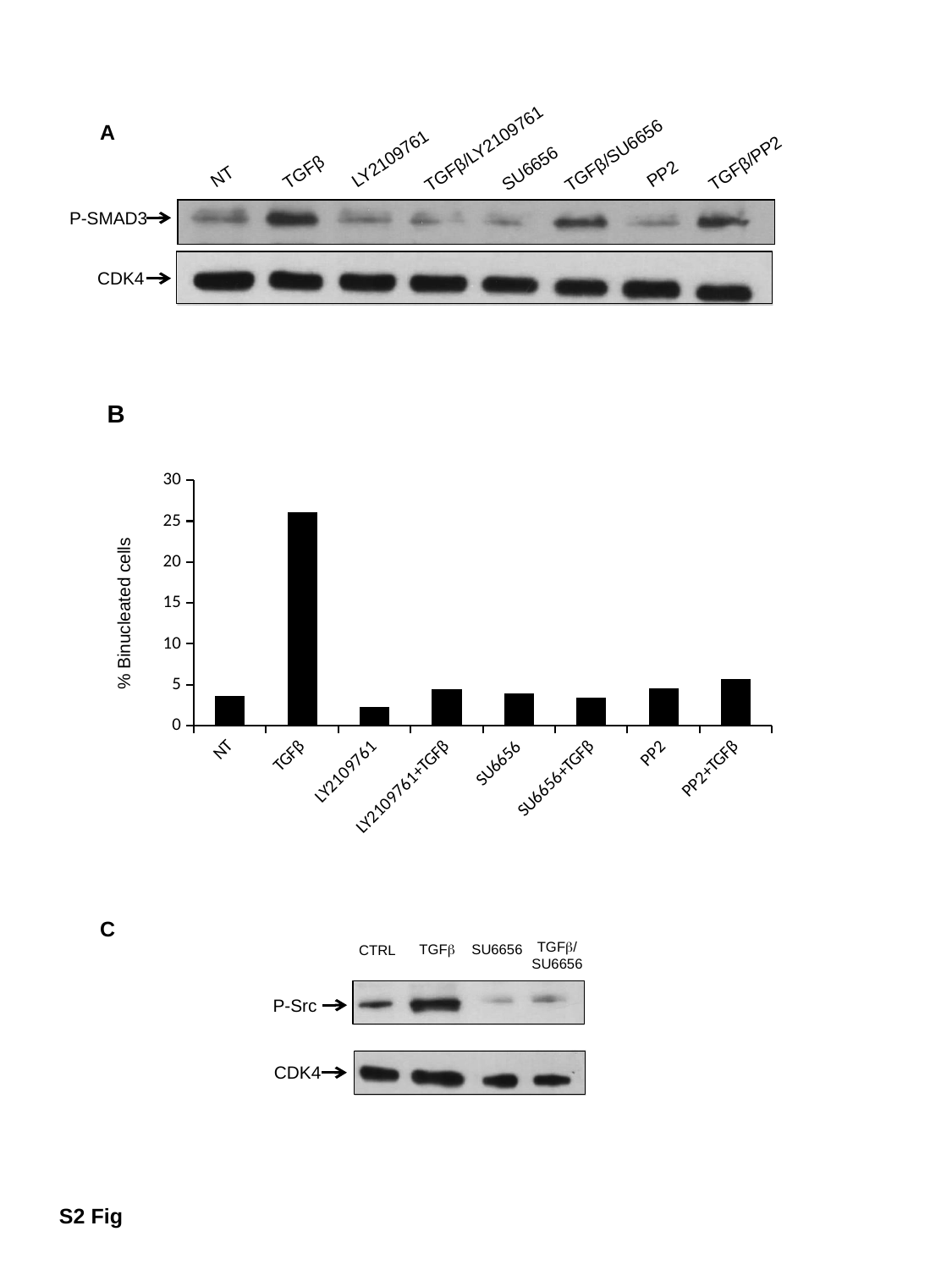

A
TGFβ/LY2109761
TGFβ/SU6656
LY2109761
TGFβ/PP2
SU6656
TGFβ
PP2
NT
P-SMAD3
CDK4
B
### Chart
| Category | |
|---|---|
| NT | 3.6 |
| TGFβ | 26.0 |
| LY2109761 | 2.2 |
| LY2109761+TGFβ | 4.4 |
| SU6656 | 3.9 |
| SU6656+TGFβ | 3.4 |
| PP2 | 4.5 |
| PP2+TGFβ | 5.6 |% Binucleated cells
C
TGFb/
SU6656
CTRL
TGFb
SU6656
P-Src
CDK4
S2 Fig
