# Supplementary material for: TGFbeta Induces Binucleation/Polyploidization in Hepatocytes through a Src-Dependent Cytokinesis Failure
Source: PLoS One. 2016 Nov 28;11(11):e0167158. doi: 10.1371/journal.pone.0167158 (PMC5125678; doi:10.1371/journal.pone.0167158)
Supplement: S3 Fig — (A) Immunofluorescence for E-Cadherin of MMH/E14 hepatocytes in the indicated experimental conditions. Arrows indicate the binucleated cells. (B) Transcriptional analysis by qRT-PCR of E-Cadherin and Snail of MMH/E14 hepatocytes in the indicated experimental conditions. Data are expressed as average values of three different experiments ± s.e.m., and plotted as ratio between treated and untreated (CTRL = 1) cells. (PPTX) [file pone.0167158.s003.pptx]

## Slide 1
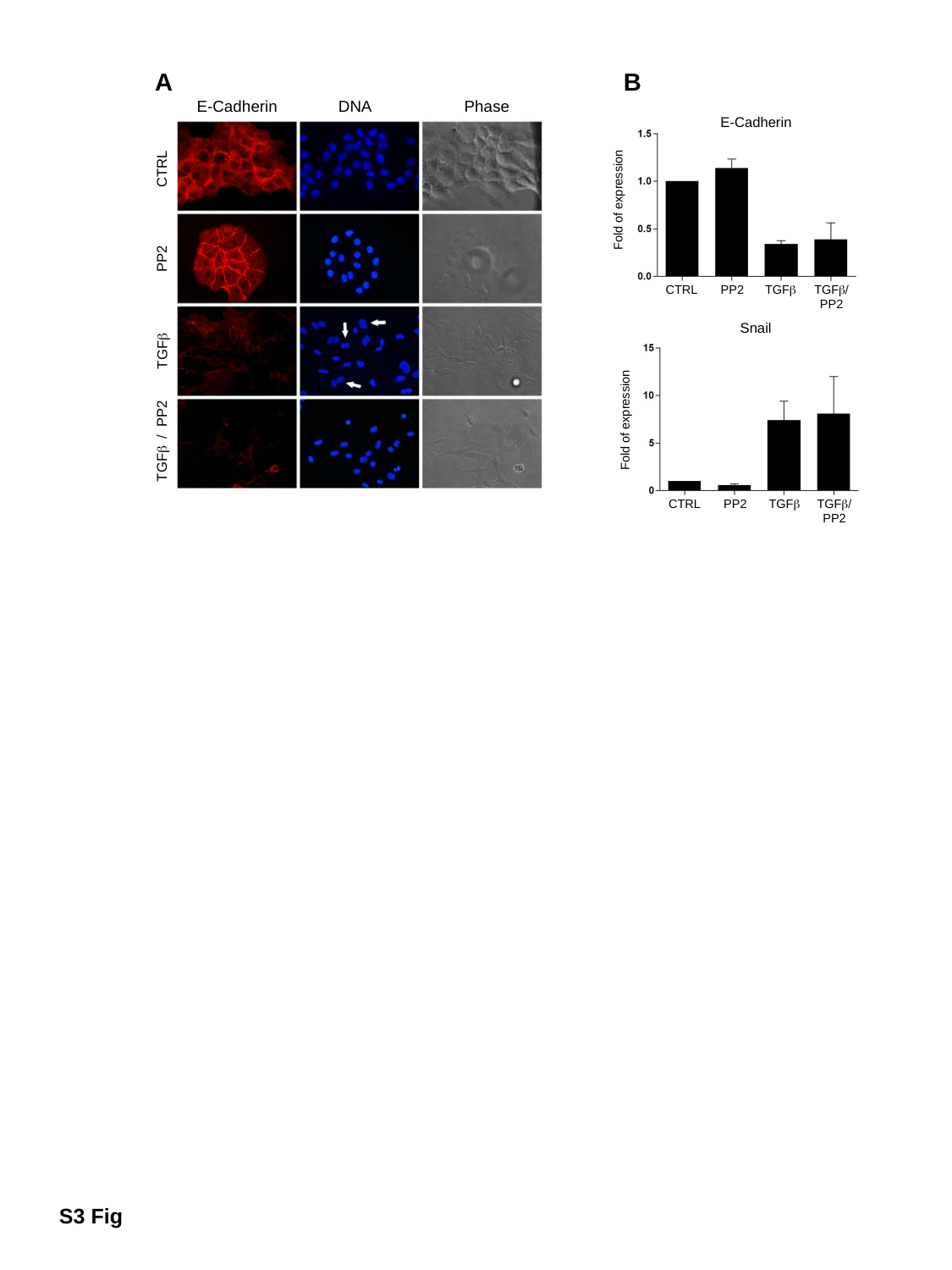

A
B
E-Cadherin
DNA
Phase
E-Cadherin
CTRL
Fold of expression
PP2
CTRL
PP2
TGFb
TGFb/
PP2
Snail
TGFb
Fold of expression
TGFb / PP2
CTRL
PP2
TGFb
TGFb/
PP2
S3 Fig
